# Supplementary material for: Differential cell autonomous responses determine the outcome of coxsackievirus infections in murine pancreatic α and β cells
Source: eLife. 2015 Jun 10;4:e06990. doi: 10.7554/eLife.06990 (PMC4480275; doi:10.7554/eLife.06990)
Supplement: Supplementary file 1. — Comparison between differentially expressed genes in granule cell neurons (compared to cortical neurons) and pancreatic α cells (compared to β cells). DOI: http://dx.doi.org/10.7554/eLife.06990.028 [file elife06990s001.docx]

**Supplementary file 1. Comparison between differentially expressed genes in granule cell neurons (compared to cortical neurons) and pancreatic α cells (compared to β cells)**

|  | **Mouse (Microarray)** | **Mouse (RNAseq)** |
| --- | --- | --- |
|  | **Fold difference of basal expression (GCN vs. CN)** | **Fold difference of basal expression (Alpha vs. Beta)** |
| **Ccl7** | 61.0 | 24.9 |
| **Ifi27** | 58.1 | 0.2 |
| **Cxcl16** | 31.9 | 6.0 |
| **Usp18** | 29.4 | 1.0 |
| **Ifit1** | 25.5 | 2.0 |
| **Ccl5** | 24.8 | 36.1 |
| **Mnda** | 24.6 | 15.8 |
| **Tlr2** | 24.1 | 1.1 |
| **Ccl4** | 19.3 | 11.3 |
| **Ifi204** | 18.1 | 21.9 |
| **Hpse** | 16.6 | 0.7 |
| **Tlr1** | 14.8 | 58.4 |
| **Cxcl1** | 14.0 | 2.7 |
| **Cxcl2** | 14.0 | 27.6 |
| **Tnf** | 13.9 | 7.0 |
| **Rsad2/Viperin** | 13.0 | 2.1 |
| **Oas1** | 12.5 | 1.6 |
| **Ifi203** | 12.4 | 8.5 |
| **Trim30** | 11.6 | 13.1 |
| **Ifi44** | 10.3 | 2.3 |
| **Irgm2** | 10.0 | 2.1 |
| **P2ry6** | 9.6 | 0.3 |
| **Atf3** | 9.5 | 6.1 |
| **Igtp** | 9.4 | 1.2 |
| **Stat1** | 9.2 | 1.2 |
| **Slc15a3** | 8.9 | 21.9 |
| **Unc93b1** | 8.0 | 2.2 |
| **Casp12** | 7.7 | 10.4 |
| **Psmb8** | 7.0 | 2.1 |
| **Tlr7** | 6.7 | 102.4 |
| **Bst2/Tetherin** | 6.6 | 2.6 |
| **Tlr4** | 6.6 | 5.5 |
| **Tlr6** | 6.5 | 11.7 |
| **Isg15** | 6.4 | 5.5 |
| **Il1b** | 6.1 | 1.6 |
| **Bcl2l14** | 5.9 | 4.1 |
| **Mx1** | 5.9 | 1.3 |
| **Ifit3** | 5.6 | 2.1 |
| **Rtp4** | 5.2 | 2.9 |
| **Irf7** | 4.7 | 1.3 |
| **Ikbke** | 4.6 | 1.1 |
| **Zbp1** | 4.5 | 7.1 |
| **Ifi47** | 4.3 | 14.2 |
| **Tgtp1** | 4.2 | 5.8 |
| **Cxcl10** | 3.5 | 1.7 |
| **Trim25** | 3.4 | 1.0 |
| **Ddx58/Rig-I** | 3.4 | 2.0 |
| **Cxcl12** | 3.4 | 3.0 |
| **Casp1** | 3.3 | 8.1 |
| **Ifitm3** | 3.2 | 13.6 |
| **Ifi35** | 3.0 | 1.3 |
| **Irf9** | 2.8 | 1.4 |
| **Ifih1/Mda5** | 2.8 | 7.8 |
| **Phf15** | 2.5 | 1.1 |
| **Nod2** | 2.4 | 4.6 |
| **Tlr5** | 2.4 | 1.6 |
| **Nod1** | 2.4 | 0.9 |
| **Stat2** | 2.4 | 1.6 |
| **Jak1** | 2.1 | 0.8 |
| **Isg20** | 2.0 | 0.8 |
| **Pkr** | 2.0 | 1.6 |
| **Rnasel** | 0.4 | 2.3 |
| **Iigp1/Iigp1b** | 0.4 | 2.4 |

Gene expression was obtained from published data of microarray analysis of mouse brain cells [19] or RNAseq of purified mouse α and β cells [33]. Only genes present in both conditions are shown.
