# Supplementary material for: Differential cell autonomous responses determine the outcome of coxsackievirus infections in murine pancreatic α and β cells
Source: eLife. 2015 Jun 10;4:e06990. doi: 10.7554/eLife.06990 (PMC4480275; doi:10.7554/eLife.06990)
Supplement: Supplementary file 2. — List of primers used in the study. DOI: http://dx.doi.org/10.7554/eLife.06990.029 [file elife06990s002.docx]

**Supplementary file 2. List of primers used in the study**

| **genes** | **Forward** | **Reverse** |
| --- | --- | --- |
|  | **Sequence (5'-3')** | **Sequence (5'-3')** |
| **GAPDH** | AGTTCAACGGCACAGTCAAG | TACTCAGCACCAGCATCACC |
| **CVB5_VP1** | TTACGGCGAAAGCTTGAGAT | GTGGACGTCTGCCAACTGTA |
| **iNOS** | GGGAGCCAGAGCAGTACAAG | GGCTGGACTTCTCACTCTGC |
| **CXCL10** | GCAAGTCTATCCTGTCCGCAT | GGGTAAAGGGAGGTGGAGAGA |
| **CCL2** | TAGCATCCACGTGCTGTCTC | TGCTGCTGGTGATTCTCTTG |
| **IFNα** | TCTTCACACTCCTGGCACAA | TTGAGCCTTCTGGATCTGCT |
| **IFNβ** | GCCTTTGCCATTCAAG | AGACAGAGCTTCTGGA |
| **STAT1** | TGAGTTCCGACACCTGCAACTGAA | AGGTGGTCTCAAGGTCAATCACCA |
| **CAR** | TGTCCGATATTGGCACTTACC | CGAAGCACCTTGTACCTGAA |
| **DAF** | GTGCTCCACTGAGACTGAATTA | CCTGGCCGACACTCAAAT |
| **MDA5** | TGTCTTGGQCQCTTGCTTCG | TGCTGAGAAGGATTGTGCAG |
| **RIG-1** | AAAGCCAGAGACCAAGACCA | TATCTCCGCTGGCTCTGAAT |
| **PKR** | ACCTACTGTGCGGTTTCTTG | ACACAAATGTCCTGGCATCT |
| **TLR-3** | CTGAGTTTGAAGCGAGCATTTAC | TCGTCCATGTTGAGATGTTCC |
| **MX1** | GTAAGACACGACCCTCTCAAAG | GGACATCCTCCTGACAAAGAAA |
| **Viperin** | TTCAGTGGGTTTGATGAGAAGA | ACTCCTCGTAGCTGGTTAGA |
| **Tetherin** | CGCATCAAGGAACTTGAGAATAAG | CACCTGCACTGTGGTAGAAA |
| **USP18** | TGTTTGTTGGGTGACCTGGAAGGAT | TGCAGCAGATGTGGGTACAGGAGAG |
| **Isg15** | GAAGCAGGTTGCTCAGAAGA | TGATAAGGGCAACACCATCC |
| **OAS1** | TTCGGACGGTCTTGGAATTAG | GCTGCCTTCTCAGGTACTTT |
| **MAVS** | CCACCTGTGTCAGCACTAAA | CTGATGGGCAACTTGGAAGA |
| **C3** | AGTGCAGAAACGCCCTAAA | CGTGTCCTTCCCAATGATGTA |
| **GBF1** | GCTGGACACAGTGATGAAGA | CAAGTCCTGACTGACCTGTAAA |
| **ATF3** | GCTGGAGTCAGTCACCATCA | ACACTTGGCAGCAGCAATTT |
| **IRF7** | GGCAAGTGCAAGGTGTACTG | GCCCAAAACCCAGGTAGA |
| **CCL5** | CCAGAGAAGAAGTGGGTTCA | AGCAAGCAATGACAGGAAAG |
| **IL-1β** | ACAGACCCCAAAAGATTAG | AATGAGTGACACTGCCTTC |
| **ARX** | GCCCGAGTGCAAGAGTAAA | GCTCCCAGAAGCCTCATTT |
| **GCG** | GTCCCACAAGGCAGA | CTCCTCCGTGTCTTGAG |
| **INS2** | TGTGGTTCTCACTTGGTGGA | CTCCAGTTGTGCCACTTGTG |
| **PDX-1** | GGTATAGCCAGCGAGATGCT | GCTCCCAGAAGCCTCATTT |
